# Development and initial psychometric evaluation of the Body Image Matrix of Thinness and Muscularity – Female Bodies

**DOI:** 10.1186/s40337-020-00345-w

**Published:** 2020-12-01

**Authors:** Beate Steinfeld, Andrea S. Hartmann, Manuel Waldorf, Silja Vocks

**Affiliations:** Department of Clinical Psychology and Psychotherapy, Osnabrück University, Knollstraße 15, 49088 Osnabrück, Germany

**Keywords:** Body image, Body fat, Eating disorders, Figure rating scale, Women, Muscularity

## Abstract

**Background:**

Despite evidence that thinness and muscularity are part of the female body ideal, there is not yet a reliable figure rating scale measuring the body image of women which includes both of these dimensions. To overcome this shortcoming, the Body Image Matrix of Thinness and Muscularity - Female Bodies (BIMTM-FB) was developed.

**Methods:**

The objective of this study is to analyze the psychometric properties of this measure. *N* = 607 non-clinical women and *N* = 32 women with eating disorders answered the BIMTM-FB as well as instruments assessing eating disorder symptoms and body image disturbance in order to test the convergent validity of the BIMTM-FB. To assess test-retest reliability, a two-week interval was determined.

**Results:**

The results indicated that the body-fat dimension of the BIMTM-FB correlates significantly with the Contour Drawing Rating-Scale, the Drive for Leanness Scale (DLS) and the Body Appreciation Scale, while the muscularity dimension of the BIMTM-FB was significantly associated with the DLS and the Drive for Muscularity Scale, proving the convergent validity of the BIMTM-FB. High coefficients of test-retest reliability were found. Moreover, the BIMTM-FB differentiated between the clinical sample and the non-clinical controls.

**Conclusions:**

The BIMTM-FB is a figure rating scale assessing both thinness and muscularity as part of the female body ideal. Due to its high reliability and validity, the BIMTM-FB can be recommended in research and practice.

**Supplementary Information:**

The online version contains supplementary material available at 10.1186/s40337-020-00345-w.

## Plain English summery

The article introduces a new developed psychometric measure of the body image of females including both thinness and muscularity (the BIMTM-FB). It assesses women’s estimations of their actual, felt and ideal body. Pictures of realistic-looking standardized female bodies are used as stimuli. They are varied systematically regarding thinness, leanness and muscularity. The measure includes the extreme bodyshapes: very thin to very fat and very unmuscular to very muscular. Thus also severely under- or overweight patients are able to identify themselves with the figures. The application is quick and almost language-free. In research and clinical practice, the BIMTM-FB can assess women’s body image ideals, whether they meet their body image ideals, and whether body ideals might be altered through therapeutic interventions. Results indicate that women who appreciate their own body tend to have a body ideal with a higher body fat percentage. Furthermore, women with eating disorders compared to the non-clinical sample showed a significant preference for severely underweight bodies and are willing to spend significantly more time and money in order to achieve their ideal body shape.

## Background

Body image is defined as the “picture we have in our minds of the size, shape and form of our bodies; and our feelings concerning these characteristics …” ([1], p. 20). Body image disturbance is associated with psychosocial impairment and is assumed to be a crucial factor in the development [2, 3], maintenance [4] and relapse [5] of eating disorders and body dysmorphic disorder [6, 7]. Moreover, it is a core symptom of and diagnostic criterion for eating disorders [2, 4] and body dysmorphic disorder [6, 7], as described in the DSM-5 [8].

Body image disturbance is conceptualized as a multidimensional construct. Beyond body-related perceptions, checking and avoidance behavior, it consists of thoughts, feelings and attitudes related to physical aspects of one’s body [9]. The two basic body image-related attitudes are body image evaluation and body image investment [10]. Body image evaluation refers to one’s beliefs about and appraisal of one’s appearance, such as body satisfaction or body dissatisfaction. Body image investment implies the core affect-laden importance that individuals place on their appearance, and describes the degree to which they define themselves and their self-worth based on their appearance [11]. When assessing body image disturbance, Cash [10] recommends that both body image evaluation and body image investment are considered, in order to measure not only how people evaluate their appearance, but also how important their looks are to them and their self-confidence [10].

A positive body image cannot be equated with low values in body image disturbance (Tiggemann & McCourt, 2013). Rather, a key characteristic of a positive body image appears to be body appreciation [12, 13], which is defined as accepting and respecting one’s body and holding favorable opinions toward it [14]. It is assumed that body appreciation acts as a protective factor against sociocultural appearance pressures [13, 15]. This functional quality of body appreciation was empirically supported in an experimental study by Halliwell (2013), which indicated that body appreciation protected women from negative media exposure effects. Moreover, Andrew et al. [16] found that in young women, greater body appreciation was related to lower internalization of societal ideals of attractiveness (thin-ideal internalization, [17]). According to the tripartite influence model of body image and eating disturbance, the internalization of such societal appearance standards in turn influences the development of body image disturbance [9].

In recent years, it has been acknowledged that the sociocultural ideal of physical attractiveness in women is shifting away from a very thin body ideal toward an athletic, ultra-fit body ideal [18–20]. This athletic ideal is characterized by low body fat and toned, physically fit muscles, and women have been found to rate other females’ bodies as more attractive when they are both thin and muscular as opposed to purely thin [18]. It therefore appears that the traditional gender-specific polarization in body image disturbance research – reducing the female body ideal to the drive for thinness [21] and the male body ideal to the drive for muscularity [22, 23] – is obsolete. Smolak and Murnen [24] developed the Drive for Leanness Scale and found that drive for thinness, drive for leanness and drive for muscularity can be understood as distinct components of body image. Moreover, the authors reported that while women have a significantly lower ambition to be muscular compared to men, their desire to have an athletic and toned body with physically fit muscles is as high as it is for men. Although Kelley et al. [25] found a significantly lower drive for muscularity in women than in men, 54% of the female participants nevertheless showed an elevated drive for muscularity. Furthermore, the presence of both drive for thinness and drive for muscularity was correlated with a specific profile of psychological risk factors, for instance regarding body compulsivity and body anxiety, underscoring that muscularity is part of the body ideal of women as well [26, 27].

In order to measure body image disturbance, besides self-rating scales, figure rating scales offer a complementary methodological approach [28–31]. Such scales consist of silhouettes, drawings or photographs of the human body that range from very thin to obese. In the majority of studies employing such scales, participants are asked which figure most accurately represents their current body size and which one represents their ideal body size. The difference between the current and the ideal size is called the self-ideal discrepancy [32] and can be interpreted as a measure for body shape dissatisfaction [33], thus quantifying body image evaluation [10].

The advantage of figure rating scales lies in their ease of use and their visual appeal [34]. Moreover, they can be implemented as an almost nonverbal instrument and can thus be applied transculturally as well as with illiterate patients. As they only contain two or three items, such scales are also very time- and cost-efficient. The existing figure rating instruments differ with respect to the quality of the images, i.e. silhouettes, pictures, or contours, the perspective which is presented, i.e. front or side, and the number of figures displayed. One widely used silhouette-based instrument is the Contour Drawing Rating Scale (CDRS [31];), which consists of nine silhouettes ranging from very thin to large, representing different body-fat percentages [31, 35]. Additionally, Mutale et al. [36] developed the Body Dissatisfaction Scale (BDS) as a figure rating scale with nine female and nine male computer-generated avatars which also vary regarding body fat. A further established figure rating scale is the Somatomorphic Matrix [28], which is a dimensional computerized test for men and women aiming to measure satisfaction and perceptual accuracy regarding muscularity and body fat. The matrix consists of 10 × 10 contour-drawn silhouettes [28]. Like most of the existing figure rating scales for women, the CDRS and BDS show good psychometric properties. However, the CDRS only includes the body-fat dimension [29–31], thus neglecting muscularity as an additional continuum [37]. The only widely used figure rating scale for women that includes muscularity is the Somatomorphic Matrix [28], but this suffers from the important shortcoming of poor test-retest reliability [38]. Thus, to the best of our knowledge, there is no figure rating scale for women which includes the dimensions of thinness as well as leanness and muscularity and also shows satisfactory psychometric properties.

Another substantial weakness of the existing measures is that the matrices do not include extreme shapes e.g., extreme thinness or extreme overweight [32]. As patients who suffer from eating disorders are often located at the extremes concerning both their actual and ideal body shape (Kessler et al., 2013; Tovée et al., 2003), there is a risk that they will be unable to identify themselves with the bodies presented, leading to a reduced variance and invalid results. A further common deficit of existing figure rating scales is that the line-drawn stimuli appear unrealistic, thus hindering participants’ identification with the drawings and resulting in a lack of ecological validity [30]. For some figure rating scales, ecological validity is also limited by potentially distracting details like faces or clothes [28, 30, 31, 37]. Others present bodies with inconsistent postures [39] or inconsistent body proportions, such as different length of legs [30, 31]. As such, the bodies also differ in characteristics other than body fat or muscularity, which may have an effect on participants’ ratings.

**Fig. 1:**
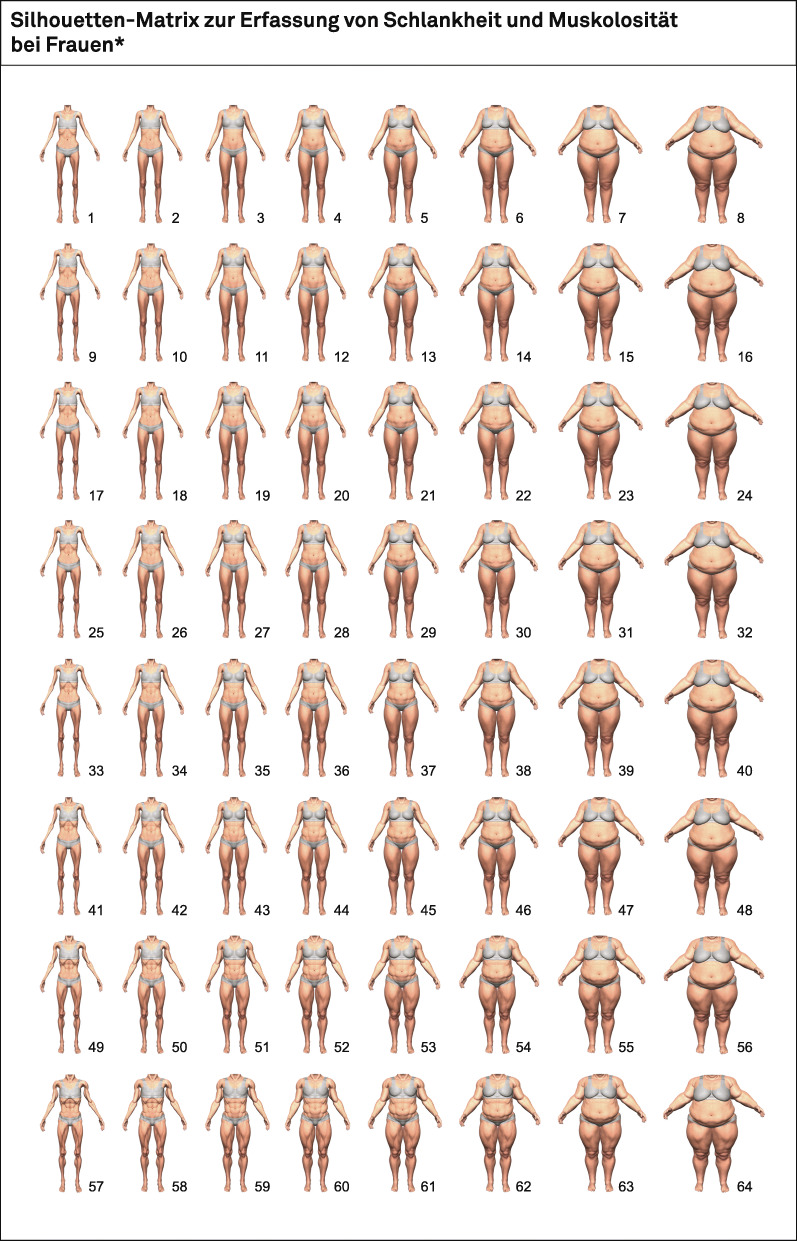
The Body Image Matrix of Thinness and Muscularity – Female Bodies

To overcome these limitations, we developed the Body Image Matrix of Thinness and Muscularity – Female Bodies (BIMTM-FB) as an instrument that captures the two dimensions body fat and muscularity in a finely graduated manner. As stimuli, this new figure rating scale for women uses realistic-looking bodies that vary systematically regarding thinness, leanness and muscularity, including the extremes very thin to very fat and very unmuscular to very muscular. The BIMTM-FB is shown in Fig. 1.

The aim of the present study was to assess the validity and test-retest reliability of the BIMTM-FB in a random sample of adult women as well as in a clinical sample of women with eating disorders. We expected the BIMTM-FB to demonstrate convergent validity through correlation of the scores with several other body image-related indices. More specifically, we expected the BIMTM-FB to be strongly and positively associated with another validated figure rating scale, the CDRS. Thus, we hypothesized that participants’ ratings regarding their actual, felt and ideal figure on the body-fat dimension of the BIMTM-FB would be associated with their corresponding ratings on the CDRS. Furthermore, we predicted a positive correlation between participants’ BMI and their reported actual body size based on the body-fat dimension of the BIMTM-FB. Moreover, we anticipated an inverse relationship of the rated body ideal on the body-fat dimension of the BIMTM-FB with the drive for leanness, insofar as women with a high drive for leanness would choose body shapes with less body fat as their ideal shape and vice versa [24]. In addition, as previous research indicated that a higher level of body appreciation is associated with a lower level of thin-ideal internalization [16], it was expected that a positive body image, measured with the Body Appreciation Scale (BAS-2, [14]), would show a moderate positive relationship with the rated body ideal on the body-fat dimension of the BIMTM-FB. In other words, we expected a higher level of body appreciation to be associated with a body ideal characterized by a higher percentage of body fat. Furthermore, as patients with anorexia and bulimia nervosa have, by definition, a thin body ideal [8], we hypothesized that the rated body ideal on the BIMTM-FB-BF would be able to differentiate between a clinical sample of patients with eating disorders and a non-clinical control sample. Regarding the muscularity dimension of the BIMTM-FB, we assumed that the rated body ideal on the muscle dimension of the BIMTM-FB would be positively correlated with participants’ drive for muscularity [40, 41]. Moreover, we anticipated a moderate positive correlation between participants’ body ideal and their drive for leanness. We further expected that the BIMTM-FB scores on both dimensions would be stable over a two-week period, thus confirming the test-retest reliability of the measure. Finally, we expected that women with eating disorders would differ significantly from non-clinical controls with respect to their body image investment [10], meaning that patients with eating disorders would be willing to invest significantly more of their resources in order to reach their body ideal.

## Method

### Participants

Inclusion criteria for the present study were female sex and a minimum age of 18 years. The sample comprised *N* = 639 female participants who were divided into four subsamples. The first two subsamples (S1 and S2) consisted of non-clinical control participants. S1 (*n* = 398) completed an online version of the questionnaire battery, while S2 (*n* = 179) completed a paper-and-pencil version. These participants were recruited at the University of Osnabrück in Germany through advertising in university classes and on notice boards. Students who completed the questionnaire battery received course credits. The online sample S1 was additionally recruited via social media, press reports and flyers. The online survey was implemented via Unipark (Questback GmbH, Berlin; Germany). A link or a QR-code led the participants to the online questionnaires. The S1 participants entered a lottery to win one of ten 20-euro online shopping vouchers. Sample characteristics of S1 and S2 as well as the means and median scores on the employed diagnostic tests are shown in Table 1.

**Table 1:** Descriptive Statistics: Sample Characteristics of S1 and S2

	S1: Online sample	S2: Paper pencil sample
	*N* = 398	*N* = 179
	Mean (SD)	Mean (SD)
Age	25,04 (6,89)	22,34 (3,78)
BMI	23,33 (4,77)	22,07 (3,62)
CDRS	Median (interquartile range)	Median (interquartile range)
Actual	5,00 (3,00)	5,00 (2,00)
Felt	6,00 (2,00)	6,00 (2,00)
Ideal	4,00 (2,00)	4,00 (2,00)
BIMTM-FB-BF	Median (interquartile range)	Median (interquartile range)
Actual	4,00 (2,00)	4,00 (1,00)
Felt	4,50 (1,00)	4,00 (1,00)
Ideal	3,00 (1,00)	3,00 (0,00)
Actual	2,00 (2,00)	1,00 (1,00)
Felt	1,00 (1,00)	1,00 (1,00)
Ideal	2,00 (2,00)	2,00 (2,00)
DLS-score (with reversed polarity)	Mdn = 3,67; IQR = 1,50	Mdn = 3,17; IQR = 1,33
	M (SD) = 3,57 (1,06)	M (SD) = 3,11 (0,99)
DMS-score (with reversed polarity, without item 10)	Mdn = 2,00; IQR = 0,86	Mdn = 1,93; IQR = 0,89
	M (SD) = 2,11 (0,71)	M (SD) = 2,02 (0,68)
DMS-subscale: Muscularity oriented body image	Mdn = 2,57; IQR = 1,29	Mdn = 2,29; IQR = 1,26
	M (SD) = 2,57 (0,96)	M (SD) = 2,46 (0,95)
DMS-subscale: Muscularity behavior	Mdn = 1,43; IQR = 0,71	Mdn = 1,43; IQR = 0,71
	M (SD) = 1,66 (0,72)	M (SD) = 1,59 (0,71)
BAS-score	Mdn = 3,5; IQR = 1,10	Mdn = 3,6; IQR = 7,31
	M (SD) = 3,36 (0,79)	M (SD) = 3,52 (0,73)

*Note. CDRS* Contour Drawing Rating Scale; *BIMTM-FB-BF* body fat dimension of the Body Image Matrix of Thinness and Muscularity – Female Bodies; *BIMTM-FB-M* muscularity dimension of the Body Image Matrix of Thinness and Muscularity – Female Bodies; *DLS* Drive for Leannes Scale; *DMS* Drive for Muscularity Scale; *BAS* Body Appreciation Scale

Subsample S3 consisted of *N* = 32 female inpatients of a cooperating clinic for eating disorders, aged between 18 and 32 years (*M* = 22.22 years, *SD* = 3.78). S3 participants completed the BIMTM-FB as a paper-and-pencil test and additionally underwent the SCID-I (Structured Clinical Interview for DSM-IV Axis I disorders), which was conducted by a clinical psychologist. Based on this, *n* = 15 women were diagnosed with bulimia nervosa (BN) and *n* = 17 with anorexia nervosa (AN) (AN-restrictive: *n* = 12, AN-binge-purging: *n* = 5). Subsample S4 comprised *n* = 30 participants of a community sample from a rural region in Northern Germany. These participants completed the BIMTM-FB as a paper-and-pencil test twice over a 14-day interval in order to assess test-retest reliability. The average age of S4 participants was *M (SD)* = 36.80 (6.92) years and their mean BMI was *M (SD)* = 28.04 (9.20) kg/m^2^. The participants of S2 and S4 completed the BIMTM-FB directly after recruitment. Participants of S4 received a second questionnaire form, which they were asked to complete after a two week-interval and to mail it back to the first author in the provided pre-paid envelope. Before beginning to answer the items, all participants were informed that they had the option to quit the survey at any time. Moreover, the content and the aim of the study, the voluntary and anonymous nature of participation, privacy protection and the duration of on average 35 min were explained. The study was approved by the local ethics committee.

### Measures

#### Body Appreciation Scale-2 (BAS-2)

The BAS-2 [14] encompasses 10 items assessing the appreciation of one’s own body [42]. The items assess individuals’ acceptance of their body, their favorable opinion toward their body, as well as their respect for their body. Items are rated on a 5-point Likert scale (1 = never to 5 = always). Reliability of the English-language version of the BAS-2 was Cronbach’s α = .94 [14]. Reliability for the BAS-2 in the current study was Cronbach’s α = .91. The test-retest reliability of the English version over a three-week period was *r*_tt_ = .90 [14]. In a female student sample the construct validity of the BAS-2 was supported by its strong positive correlations with appearance evaluation (*r* = .80), self-esteem (*r* = .71) and proactive coping (*r* = .39) as well as by its significant negative association with body dissatisfaction (*r* = −.73) [14].

#### Body Image Matrix of Thinness and Muscularity-Female Bodies (BIMTM-FB)

The BIMTM-FB is a figure rating scale that contains the two orthogonal dimensions body fat (BIMTM-FB-BF) and muscularity (BIMTM-FB-M), each consisting of eight figures which increase in body fat and muscularity in small increments. Thus 8 × 8 female bodies – overall 64 figures – are displayed, covering a range of body shapes from very thin to obese and very unmuscular to very muscular. The level of body fat of the figures increases successively in the horizontal direction and the level of muscularity increases in the vertical direction. Consequently, each of the 64 multicolored silhouettes has its own specification on the dimension of body fat and on the dimension of muscularity. The BIMTM-FB is shown in Fig. 1. The bodies of the BIMTM-FB were generated using the rendering Software *DAZ Studio 4.9 Pro (64-bit)*. We used a commercially available 3D model for the bodies (“Victoria 6”, based on the Genesis software platform by DAZ Productions, Inc., see https://www.daz3d.com/victoria-6), which lends a skin texture to the underlying polygonal base mesh and allows for realistic-looking body morphs. In order to minimize distraction and to enhance the focus on the body and its shape, standardized postures of the torso and extremities were chosen. Moreover, the figures end below the face and are depicted wearing discreet underwear. The items of the BIMTM-FB are identical to those of the CDRS [31, 43] in order to optimize the comparability of the two instruments. Figures are numbered consecutively from 1 to 64, enabling participants to write down the number of the figures which most closely resemble their actual (“How do you actually look?”), their felt (“How do you feel you look?”) and their ideal body (“How would you like to look?”). These questions were also used, for example, in studies by Vocks et al. [44] and a study by Arkenau et al. (2020).

### Body mass index (BMI)

Participants were asked to report their weight in kilograms and their height in centimeters. This information was then used to calculate their body mass index (BMI = weight in *kg* / height in *m*^*2*^). As found, for example, by Elgar et al. [45], self-reported and measured height and weight are highly correlated, although an underreporting of body weight by an average of .52 kg was noted.

### Contour Drawing Rating Scale (CDRS)

To test the convergent validity of the BIMTM-FB, the CDRS [31, 43] as a further figure rating scale was applied. Participants are asked to mark the drawing which most closely resembles their actual, their felt and their ideal body. Nine hand-drawn female figures with ascending body fat are displayed. The one-week test-retest reliability in a sample of female undergraduate students was *r*_tt_ = .78 [31]. Thompson & Gray [31] also reported significant associations of the chosen actual figure via the CDRS with the reported weight (*r* = .71) and the participants’ BMI (*r* = .59), thus confirming the concurrent validity of the CDRS [32].

### Drive for Leanness Scale (DLS)

To assess the desire to have a lean and athletic body with trained muscles, the DLS ([24, 27], Hartmann, unpublished data) was used. The six items of the DLS are rated on a 5-point response scale ranging from 1 = never to 6 = always. The internal consistency of the German version was Cronbach’s α = .78. For the English version of the DLS, in a female and male student sample, Tod et al. [46] reported correlations of drive for leanness with athletic internalization (*r* = .52), pressure to attain an ideal physique (*r* = .25), exercise frequency (*r* = .36), and dieting (*r* = .25).

### Drive for Muscularity Scale (DMS)

In order to quantify the individual desire to have a muscular body, the 15-item DMS [40, 41] was used. The items are rated on a 6-point Likert scale ranging from 1 = always to 6 = never, with higher scores indicating a lower drive for muscularity. As recommended by Waldorf et al. [41], the scores were reverse-coded and item 10, which asks about steroid use, was excluded. The internal consistency of the German version of the DMS ranges between α = .89 and .91, and the nine-day test-retest reliability lies at *r*_tt_ = .95 [41]. For a sample of female weight-trainers, Hartmann et al. found significant correlations between the DMS and drive for thinness (*r* = .31) as well as the DLS (*r* = .36).

### Eating Disorder Examination-Questionnaire (EDE-Q)

To assess the psychopathology of eating disorders, the EDE-Q [47, 48], as a more economical, questionnaire version of the Eating Disorder Examination interview (EDE [49]), was applied. All 22 items of the EDE-Q refer to the past 28 days and are answered on a 7-point rating scale. Items are subdivided into the four scales “Restraint” (5 items), “Eating Concern” (5 items), “Weight Concern” (5 items) and “Shape Concern” (8 items). Internal consistency for the subscales ranges between Cronbach’s α = .80 and .93 for healthy controls and between Cronbach’s α = .67 and .93 for participants with an eating disorder. In a meta-analysis of 16 studies mainly examining young women, Berg et al. [50] reported an adequate convergent validity, as shown by correlation coefficients between the EDE-Q and the EDE subscales ranging from *r* = .68 to .76.

### Investment questions

In order to evaluate the body image investment [10], the ratings of the BIMTM-FB can be supplemented with the following three questions: “How many hours per day would you invest in order to reach your rated ideal body shape?”, “What percentage of your income would you invest in order to reach your rated ideal body shape?”, “How many years of your life would you invest in order to reach your rated ideal body shape?”. By applying the three investment questions in addition to the three rating questions of the BIMTM-FB, it is possible to measure the evaluative component as well as the investment component [11, 51].

### Data analysis

The data analysis was performed using IBM SPSS Statistics 24. As an ordinal scale level can be assumed for the BIMTM-FB, only nonparametric tests were used. Convergent validity and test-retest reliability were assessed by calculating correlations i.e. Goodman and Kruskal’s Gamma (γ), which has the advantage of being a tie-robust rank correlation measure [52]. To test whether the rated body ideal on the body-fat dimension as well as the body image investment indicated by the participants were able to differentiate between patients with eating disorders and non-clinical women, a Mann-Whitney *U* test was conducted as a nonparametric test. The z-value was used to calculate the effect size, as proposed by Cohen [53]. According to Cohen’s guidelines, *r* = .5 is a large effect, *r* = .3 is a medium effect and *r* = .1 is a small effect [54].

## Results

### Convergent validity

The correlation analysis that was performed in order to test the convergent validity of the BIMTM-FB revealed significant associations of the rated actual, felt and ideal figure on the body fat dimension of the BIMTM-FB with the corresponding responses to the CDRS, as shown in Table 2.

**Table 2:** Rank Correlational Analysis with Goodman and Kruskal’s Gamma (γ) of the BIMTM-FB-BF and the CDRS

Measures	Sample	CDRS Actual	CDRS Felt	CDRS Ideal
BIMTM-FB-BF				
Actual	S1	.86***		
	S2	.89***		
Felt	S1		.84***	
	S2		.89***	
Ideal	S1			.77***
	S2			.87***

*Note. BIMTM-FB-BF* body fat dimension of the Body Image Matrix of Thinness and Muscularity (with the perceived actual, felt, and ideal body); *CDRS* Contour Drawing Rating Scale; *S1* online sample; *S2* student sample (paper-pencil)

Table 3 provides a further overview of associations between the BIMTM-FB and the other convergent measures for S1 and S2. On the body-fat dimension of the BIMTM-FB, a higher score indicates a silhouette with more body fat and a lower score indicates a figure with less body fat. Correspondingly, on the muscularity dimension of the BIMTM-FB, higher scores represent a figure with a higher degree of muscularity and lower scores represent a less muscular figure. A significant positive correlation emerged between participants’ BMI and their reported actual body size on the body-fat dimension of the BIMTM-FB. Moreover, the DLS values were significantly inversely correlated with the body ideal on the body-fat dimension of the BIMTM-FB, i.e., a higher drive for leanness was associated with less body fat of the chosen figure of the BIMTM-FB. For S2, a significant positive correlation emerged between the body-fat dimension of the BIMTM-FB and the BAS, i.e., higher body appreciation was associated with more body fat of the chosen body ideal, whereas this association did not reach significance for S1. The muscularity dimension of the BIMTM-FB was positively correlated with the DMS. A moderate association was found between the two measures. In sum, the medium to high correlations indicate a substantial convergent validity of the BIMTM-FB.

**Table 3:** Rank Correlational Analysis with Goodman and Kruskal’s Gamma (γ) of BIMTM-FB and convergent measures

Measures	Sample	BMI	BAS	DLS	DMS
	S1:*N* = 398; S2:*N* = 179				
BIMTM-FB-BF					
Actual	S1	.73***	−.18***	−.18***	−.02
	S2	.81***	−.12	−.11	.06
Felt	S1	.59***	−.38***	−.09	.05
	S2	.64***	−.35***	.03	.18**
Ideal	S1	.61***	**.09**	−.38***	−.15**
	S2	.45***	.40***	−.41***	−.25**
BIMTM-FB-M					
Actual	S1	.16**	−.02	.15**	.13**
	S2	.09	.11	.15	.02
Felt	S1	.18***	−.02	.14***	.13*
	S2	.13	.15	.14	.00
Ideal	S1	.02	−.01	.29***	.33***
	S2	−.06	−.14*	.37***	.27***

*Note.* BIMTM-FB-BF = body fat dimension of the Body Image Matrix of Thinness and Muscularity (with the perceived actual, felt, and ideal body); BIMTM-FB-M = muscularity dimension of the Body Image Matrix of Thinness and Muscularity (with the perceived actual, felt, and ideal body); BMI = Body Mass Index; BAS = Body Appreciation Scale; DLS = Drive for Leanness Scale; DMS = Drive for Muscularity Scale. S1 = online-sample, S2 = student-sample (paper-pencil); * *p* < .05. ** *p* < .01. *** *p* < .001

Table 4 summarizes the results of a series of Mann-Whitney *U* tests exploring whether BIMTM-FB scores were able to distinguish participants with eating disorders from control participants. The ideal body shape discriminated significantly between the control sample and the clinical sample. Moreover, the two samples differed significantly regarding the resources they were willing to invest in order to reach their ideal body shape.

**Table 4:** T-test and Mann-Whitney-U test regarding sample characteristics and the BIMTM-FB-BF (S2 and S3)

	Control group (S2)	Clinical group (S3)	Group comparison statistics			
	*N* = 179	*N* = 32				
	Mean (SD)	Mean (SD)	t-Value	df	p	d
Age	22,18 (3,37)	22,22 (3,78)	−0,61	209	.952	0,01
BMI	22,03 (3,62)	19,04 (4,62)	4,07	208	.000	0,79
EDE-Q						
Gesamtwert	1,60 (1,18)	3,45 (1,36)	−8,09	205	.000	1,53
restraint	1,20 (1,24)	2,97 (1,69)	−5,65	37,18	.000	1,34
Eating concern	1,60 (1,27)	2,93 (1,32)	−5,41	209	.000	1,04
Weight concern	1,93 (1,42)	3,60 (1,61)	−5,97	206	.000	1,15
Shape concern	1,50 (1,20)	4,30 (1,33)	−11,97	206	.000	2,30
BIMTM-FB-BF						
	Median (interquartile range)	Median (interquartile range)	Mann–Whitney U	Z	p	r
Actual	4 (1)	3 (1)	2543,50	−1,06	.145	0,07
Felt	4 (1)	5 (1)	2206,50	−2,14	.016	0.14
Ideal	3 (0)	2 (1)	1597,50	−4,86	.000	0,27
Importance	3 (1)	4 (1,5)	2303,00	−1,67	.048	0,12
			t	df	p	d
Investment	Mean (SD)	Mean (SD)				
Hours per day	1,38 (1,62)	6,15 (6,73)	−4,01	31,65	.000	1,59
Percentage of income	8,98 (9,56)	28,97 (25,93)	−4,19	32,53	.000	1,50
Years of life	1,67 (6,32)	6,84 (10,31)	−2,71	33,90	.005	0,73

Notes: *BMI* Body Mass Index, *EDE-Q* Eating Disorder Examination-Questionnaire; *BIMTM-FB-BF* body fat dimension of the Body Image Matrix of Thinness and Muscularity-Female Bodies (with the perceived actual, felt, and ideal body); *BAS* Body Appreciation Scale; *DLS* Drive for Leanness Scale; *DMS* Drive for Muscularity Scale. *S2* student-sample (paper-pencil); *S3* sample of inpatients with eating disorders

### Test-retest reliability

The test-retest reliability over a two-week period for the body-fat dimension and the muscularity dimension was assessed. Tables 5 and 6 show the median scores on the BIMTM-FB at both time points (T1 and T2) as well as the correlation analysis.

**Table 5:** Median and Test-Retest Rank Correlational Analysis with Goodman and Kruskal’s Gamma (γ) of the BIMTM-FB-BF for T1 and T2

*BIMTM-FB-BF*	Actual	Felt	Ideal
T1: Mdn (IQR)	5 (2,00)	5 (2,00)	4 (1,00)
T2: Mdn (IQR)	5 (2,00)	5 (2,00)	4 (2,00)
γ (Correlation between T1 und T2)	1***	.99***	1***

*BIMTM-FB-BF* body fat dimension of the Body Image Matrix of Thinness and Muscularity (with the perceived actual, felt, and ideal body); *Mdn* median; *IQR* interquartile range; *S3*: *N* = 30; *** *p* < .001

**Table 6:** Median and Test-Retest Rank Correlational Analysis with Goodman and Kruskal’s Gamma (γ) of the BIMTM-FB-M for T1 and T2

*BIMTM-FB-M*	Actual	Felt	Ideal
T1: Mdn (IQR)	2,00 (2,00)	2,00 (3,00)	3,00 (3,00)
T2: Mdn (IQR)	2,00 (3,00)	2,00 (3,00)	3,00 (2,00)
γ (Correlation Between T1 und T2)	.99***	.99***	.98***

*Note*. *BIMTM-FB-M* muscularity dimension of the Body Image Matrix of Thinness and Muscularity (with the perceived actual, felt, and ideal body); *Mdn* median; *IQR* interquartile range; *S3* N = 30; *** *p* < .001

## Discussion

The present study aimed to psychometrically test the recently developed figure rating scale BIMTM-FB, which uses digitally created body depictions including extreme body shapes on the two dimensions thinness and muscularity. The BIMTM-FB consists of detailed, realistic female body figures and assesses respondents’ estimations of their actual, felt and ideal body, while differentiating between levels of body fat and muscularity.

The convergent validity of the body-fat dimension of the BIMTM-FB was supported by its strong positive correlation with the CDRS. Moreover, it was further supported by the significant correlation between participants’ BMI and their estimations of their actual body on the body-fat dimension of the BIMTM-FB. Furthermore, the moderate negative association of the rated body ideal with the drive for leanness points in the same direction, demonstrating that a desire to have an athletic and toned body with physically fit muscles is associated with choosing body shapes with less body fat as an ideal shape and vice versa. The moderate positive association between the muscularity dimension of the BIMTM-FB and the DMS also confirms the convergent validity. The inconsistent results of the online sample (S1) and the paper-and-pencil sample (S2) regarding the correlation between the BAS and the body ideal ratings on the body-fat dimension underline that more research needs to be done in order to clarify the link between body appreciation and the body ideal [15]. Nevertheless, the significant positive correlation between body ideal ratings on the body-fat dimension in the paper-and-pencil sample (S2) and the BAS indicates that as a trend, participants who appreciate their own body tend to have a body ideal with a higher body fat percentage. This confirms previous studies, which found that a positive body image is associated with low internalization of sociocultural appearance standards [14]. Thus, body appreciation might be one of the psychological factors that moderate an individual’s degree of vulnerability to sociocultural pressure [55]. This finding underlines the need to understand the characteristics of a functional body image and – especially for clinical practice – how it can be acquired. The BIMTM-FB can be useful to assess the effect of viewing body positive content on women’s body ideal. Such content might take the form of body positive posts on social media, with the aim of challenging mainstream beauty ideals and encouraging appreciation and acceptance of all body types [56, 57].

Furthermore, the BIMTM-FB was able to differentiate between females with and without eating disorders, with the two groups varying substantially in terms of their ideal body shape. Consistent with existing studies, we found that women with eating disorders showed a significant preference for severely underweight bodies as compared to the rated body ideal of the non-clinical sample [58, 59]. These findings emphasize the importance to include underweight bodies in figure rating scales in order to also cover the very thin body ideal of women with eating disorders, thus underscoring a strength of the BIMTM-FB.

Besides body image evaluation, participants with eating disorders were also distinguishable from control participants with respect to body image investment, as assessed through the Investment Questions. The women with eating disorders indicated that they would be willing to spend significantly more time and money in order to achieve their ideal body shape, as compared to the women in the control group. For instance, on average, eating disorder patients were willing to relinquish over six hours per day in order to reach their body ideal, as compared to the average of one hour per day reported by the non-clinical sample. These results confirm the association between body image investment and disturbed eating attitudes reported in previous research [51]. Furthermore, they support Cash’s [10] recommendation to not solely consider how individuals evaluate their appearance, but also to take into account how important their appearance is to them.

In line with our expectations, a high test-retest reliability of the BIMTM-FB over a two-week interval was confirmed, indicating the temporal stability of the BIMTM-FB ratings. This corresponds with the findings of test-retest reliability from other figure rating scales (e.g., CDRS, [31]) and marks an advantage over the Somatomorphic Matrix, which demonstrated low test-retest reliability [38]. In sum, the results indicate that the BIMTM-FB has good convergent validity as well as high test-retest reliability. Moreover, the present findings suggest that the BIMTM-FB is able to differentiate between participants with eating disorders and a non-clinical sample.

While the present study was able to provide evidence of the validity and reliability of a newly developed figure rating scale, some shortcomings need to be mentioned. First, the bodies that are presented in the BIMTM-FB are of Caucasian skin pigmentation. This may limit the identification of women from non-Caucasian populations with the presented figures, in turn impairing the ecological validity of the BIMTM-FB for these groups [60]. To overcome this limitation, the development of BIMTM-FB versions for women from different ethnic backgrounds might be considered. Second, the pictured body figures of the BIMTM-FB cannot be directly associated with anthropometric data such as BMI, fat-free mass index and body fat percentage. Thus, only if independent raters compare the individual’s actual body shape with the corresponding rating on the BIMTM-FB can the perceptual body image disturbance be measured. In future studies, this limitation can be overcome by subsequently linking anthropometric data to each body figure of the BIMTM-FB, e.g. by identifying a female model for each body figure of the BIMTM-FB that corresponds to the figure’s shape and registering the model’s BMI, fat-free mass index and body fat percentage. Third, future psychometric studies of the BIMTM-FB should include different samples that are especially vulnerable for body image disturbances. For instance, studies should aim to test the psychometric properties of the BIMTM-FB for other clinical groups, such as females with binge eating disorder, body dysmorphic disorder and its subtype muscle dysmorphia, as well as other mental disorders to which body image disturbance is relevant, like posttraumatic stress disorder, borderline personality disorder, and social anxiety disorder [61–64]. Fourth, the BMI that was used in the present study to test the convergent validity of the rated actual body size on the body-fat dimension of the BIMTM-FB was not objectively measured. Instead, we relied on participants’ disclosure of their weight and height. In future studies, the BMI should be measured objectively i.e. by weighing and measuring participants under standardized conditions.

## Conclusion

Overall, the BIMTM-FB displayed good psychometric properties as a measure of the body image of females including both thinness and muscularity. The artificial but realistic-looking standardized bodies of the BIMTM-FB offer high ecological validity. As the BIMTM-FB is quick to answer and almost language-free, it can be easily applied in research and clinical practice in various language areas and – after adjustment and validation – for intercultural comparisons. Moreover, the BIMTM-FB can be administered even in the case of illiteracy. Thus, in future research, the BIMTM-FB could be a useful addition to established body image measures in order to examine body image ideals or assess whether women’s actual body corresponds to their ideals in terms of body fat and muscularity. Another possible research approach is to test the sensitivity to change of the BIMTM-FB, exploring whether it can be used as a state or trait measure. Given a high sensitivity to change of the BIMTM-FB, as a pre-post measure, it could test whether rigidly held body ideals might be altered through therapeutic interventions. Conversely, the effect of media exposure on the body image ideal regarding thinness and muscularity could also be examined with the BIMTM-FB. Besides, as the BIMTM-FB also encompasses the severely thin, severely overweight and very muscular body shapes, it can be especially useful in clinical research and practice when working with extremely thin, obese or muscular subjects, e.g. with patients with anorexia nervosa or binge eating disorder.

## Supplementary Information

**Additional file 1.**

## Data Availability

The datasets used and analysed during the current study are available from the corresponding author on reasonable request.

## Abbreviations

BAS-2: Body Appreciation Scale-2
BIMTM-FB: The Body Image Matrix of Thinness and Muscularity – Female Bodies
BMI: Body Mass Index
CDRS: Contour Drawing Rating Scale
DLS: Drive for Leanness Scale
DMS: Drive for Muscularity Scale
EDE-Q: Eating Disorder Examination-Questionnaire
SCID-I: Structured Clinical Interview for DSM-IV Axis I disorders
S1 to S4: Subsample 1 to Subsample 4
