# Supplementary material for: Development and initial psychometric evaluation of the Body Image Matrix of Thinness and Muscularity – Female Bodies
Source: J Eat Disord. 2020 Dec 1;8:75. doi: 10.1186/s40337-020-00345-w (PMC7709434; doi:10.1186/s40337-020-00345-w)
Supplement: Supplementary file 1 — Additional file 1. [file 40337_2020_345_MOESM1_ESM.pdf]

Silhouetten-Matrix zur Erfassung von Schlankheit und Muskolosität bei Frauen*

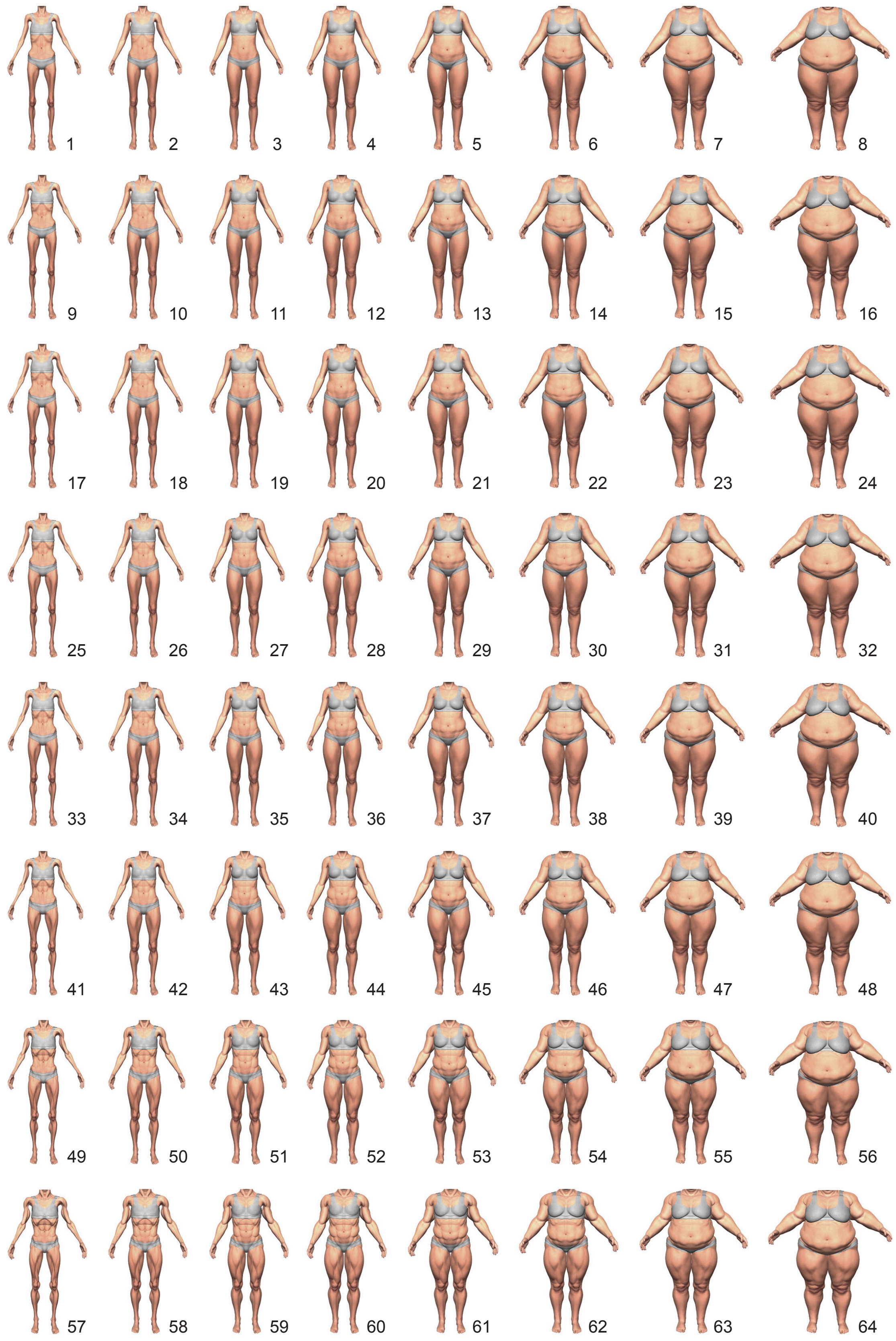

*© Steinfeld, Hartmann, Waldorf und Vocks (2017, Mai) sowie Steinfeld, Hartmann, Waldorf und Vocks (in Vorb.).
Abdruck erfolgt mit Genehmigung der Autoren.
